# The Practice Acts in Texas

**Published:** 1896-01

**Authors:** 


THE PRACTICE ACTS IN TEXAS.—So general is the misapprehension as to the requirements to practice in Texas, that exists on the part of the medical profession abroad, and even on the part of the various State boards, that we are induced to present herewith a summary of the laws now in force with reference to practicing medicine. They are ambiguous and unsatisfactory; in fact, they are almost inoperative, by reason of so many and so glaring defects. The requirements simply amount to nothing; for while the mere possession of a diploma does not give license to practice, as is generally believed abroad, and as has repeatedly been asserted by the medical press of other States,—whose editors ought to know better,—the mere act of having it registered does. Any one holding a diploma, it matters not whether issued to him or to another—it is no one's duty to ascertain if the holder be the rightful owner; it matters not if it be bought, or whence or by whom issued, save that it must be from a chartered "institution"—can practice medicine in Texas without molestation, compliance with the simple requirement of having a record made of the document in the office of the clerk of the court in the county where it is proposed to practice, making the holder of the diploma a "legal practitioner," and he can not be prosecuted for the illegal practice of medicine. Yet, strange to say, unless, in addition to having the diploma recorded, the party appear before a district board of medical examiners—whether in that district or another makes no difference—and, passing an examination, procures a certificate (for which he must pay fifteen dollars), he can not lawfully contract to practice, and can not, by suit, collect any fee for services. It will be seen that there are numerous exceptions to the operation of this law: for instance, those who had practiced five consecutive years prior to 1875, etc.

Notwithstanding what we have just said about being examined and furnished with a certificate, a majority of those now practicing in Texas, so far as we have been enabled to learn, have disregarded this latter requirement, and, having registered, they are peacefully pursuing their avocation, and no one can disturb them. In fact, until Judge King rendered the remarkable decision just quoted,—i. e., that a holder of a registered diploma must be examined by a board before he can practice for pay, i. e., collect by law for his services,—we believe that the majority of doctors were in ignorance of the existence of such requirement on the statutes. Kennedy,* it seems, although a graduate of a proper school, failed even to record his diploma; and as to the latter requirement, he put in the plea that there was no district board in his district. Kennedy was practicing right straight along, and making money. No questions were asked; he was in fellowship in all the societies, and was at the time, we believe, a professor in the Texas School of Pharmacy in the Medical Department of the University of Texas. Yet, when he sued a man for a fee, the plea was entered that Kennedy was not a legal practitioner, and not entitled by law to charge and collect for services as a physician, and the plea was supported. The judge held that the failure of the judge to appoint a board of examiners was no justification, on Kennedy's part, for failing to be examined by a board.

For years, as is well known, the State Medical Association has endeavored to secure the passage of a better law; but every effort has been ridiculed and defeated.

The articles and sections bearing upon the subject of practice are published herewith, and will be read with interest.

We want to say,—and say it loud,—and we have repeatedly said it,—for the information of all concerned, and especially for the information of secretaries of State boards, both of health and of examiners, that *there is no State Board of Health*, nor State Board of Medical Examiners in Texas. The head of the Quarantine Department is the State Health Officer, and he has general supervision over contagious disease wherever it may appear in the State; but as regards ordinary health matters and sanitation, local health officers perform the duties, and excepting that mentioned, there is no State health organization.

There are, or rather, it is made the duty of the several district judges to appoint, boards of medical examiners in each district; but as there is no penalty attached for failure to so appoint, in many districts there is no board.

The JOURNAL receives scores of letters asking about the laws governing practice in Texas. Those containing stamps are answered. In his capacity as Secretary of the Quarantine or Public Health Department, the editor gets also numerous letters on the same subject, addressed to the Secretary State Board of health. Hence this notice.

---

*See report following this.

As bearing upon and further elucidating the subject of the practice act in Texas, and for the purpose of general information, the JOURNAL reproduces the decision in the famous Kennedy case, also.

MEDICAL LAWS OF TEXAS.

The following is a copy of those articles of the laws in Texas bearing upon the subject of the practice of medicine:

*An Act to Regulate the Practice of Medicine in the State of Texas, Revised Statutes, 1879.*

Article 3626. Said board of medical examiners [appointed in each judicial district by the district judge] shall be composed of not less than three practicing physicians of known ability, and who are graduates of some medical college recognized by the American Medical Association, and who are residents of the district for which they are appointed.

Art. 3629. Said board shall meet regularly semi-annually, at some central point in their respective districts, to conduct examinations and grant certificates, as hereinafter provided; and they shall give at least one month's notice of the time and place of [each] meeting, by publication in at least one newspaper published in the district in which such meeting is to be held.

Art. 3632. It shall be the duty of the board to examine thoroughly all applicants for certificates of qualification to practice medicine in any of the branches or departments, whether such applicants are furnished with medical diplomas or not, upon the following subjects, to-wit: Anatomy, physiology, pathological anatomy and pathology, surgery, obstetrics, and chemistry; but no preference shall be given to any school of medicine.

Art. 3633. When the board shall be satisfied as to the qualifications of an applicant, they shall grant to him a certificate to that effect, which certificate shall entitle the person to whom granted to practice in any county when the same has been recorded as required by article 3635.

Art. 3634. Any two of the members of such board of examiners may grant a certificate of qualification to an applicant, and any member of said board shall have authority to grant a temporary certificate to an applicant, upon examination, until the regular meeting of the board, at which time the temporary certificate shall cease to be of force.

Art. 3635. The certificate provided for in the two preceding articles shall, before the person to whom it was granted is entitled to practice by virtue thereof, be recorded in the office of the clerk of the district court of the county in which such practitioner may reside or sojourn. * * *

Art. 3637. The provisions of this title shall not apply to the following persons:

1. To those who may have already qualified for the practice of medicine under an act entitled "An act to regulate the practice of medicine," passed May 16, 1873.

2. To those who have been regularly engaged in the general practice of medicine in this State in any of its branches or departments, for a period of five years prior to the first day of January, 1875.

3. To females who follow the practice of midwifery strictly as such.

Art. 3638. No person except those named in the preceding article [3637] shall be permitted to practice medicine in any of its branches or departments without first having obtained and recorded a certificate of qualification from some authorized board of medical examiners as hereinbefore provided, and any person offending shall be punished as provided in the penal code.

The following are the articles of the penal code referred to in article 3638:

Article 396. If any person shall practice for pay, or as a regular practitioner, medicine in this State, in any of its branches or departments, or offer or attempt to practice without first having obtained a certificate of professional qualification from some authorized board of medical examiners, or without having a diploma from some accredited medical college, chartered by the legislature of the State or its authority, in which the same is situated, he shall be punished by fine of not less than fifty nor more than five hundred dollars.

Art. 398. If any person shall hereafter engage in the practice of medicine, in any of its branches or departments, for pay or as a regular practitioner, without having first filed for record with the clerk of the district court of the county in which such person may reside or sojourn, a certificate from some authorized board of medical examiners, or a diploma from some accredited medical college, he shall be punished as prescribed in article 396.

---

The following is the full text of Judge Fly's decision in the Kennedy case, as taken from the Texas Civil Appeals Reports, Vol. 6, 1894:

### JAMES KENNEDY V. H. SCHULTZ.
### No. 226.

1. PHYSICIAN—CONTRACT FOR SERVICES VOID WITHOUT CERTIFICATE FROM BOARD OF EXAMINERS.—A physician can not recover for services rendered as such without procuring a certificate permitting him to practice medicine from the Board of Medical Examiners, and having the same recorded. The petition should allege these facts, or show the exception.

2. STATUTES CONSTITUTIONAL.—Articles 3625 to 3638, Revised Statutes, prescribing the qualifications of practitioners of medicine, are constitutional.

3. JUDICIAL NOTICE.—The court can not take judicial cognizance of whether the "American Medical Association" is composed entirely of adherents to the allopathic school of medicine.

Appeal from Bexar. Tried below before Hon. W. W. King. S. M. Ellis, for appellant; Leo Tarleton and Geo. C. Altgelt, for appellee.

FLY, ASSOCIATE JUSTICE—This suit was instituted by appellant on January 4, 1892, to recover of appellee the sum of $1200 for services rendered as a physician. It was alleged in the amended petition, that the plaintiff was a physician in Bexar county from May 1, 1890, to time of filing amendment, a graduate holding a diploma from a reputable American medical college, recognized as such by the American Medical Association; that he had never resided in any county in Texas but Bexar, and never practiced his profession in any other county in Texas; that there had not since May 1, 1890, been a duly appointed and qualified board of examiners in Bexar county, and if such board or boards were ever appointed by either of the District Courts, that none of the members had ever qualified, and for that reason it had been impossible for plaintiff to obtain a certificate as required by law, and to have the same registered. The amended petition was specially excepted to, because it appeared from the face of it that the services rendered by appellant were in violation of the civil and criminal statutes of Texas, because appellant had not caused to be recorded the certificate permitting him to practice medicine or surgery. The exception was sustained, and appellant having refused to amend, judgment was rendered for appellee.

The only question to be determined is, was the demurrer properly sustained? The reasons, as presented by the three assignments of error copied into the brief of appellant, are, first, that the title 73, article 3625 to article 3638 inclusive, are unconstitutional, because they do not impose the same obligations or burdens equally and uniformly upon the same class citizens, because the same are in conflict with that article in the State Constitution which declares, "The Legislature may pass laws prescribing the qualifications of practitioners of medicine in this State, and to punish persons for malpractice, but no preference shall ever be given by law to any school of medicine;" second, that the court erred in holding that the services rendered by appellant were in violation of the civil and criminal laws of Texas; and third, that the court erred in sustaining the demurrer, because the petition showed that there was no medical board in the judicial district in which appellant resided.

There can be no question of the constitutionality of the statute. On the question involving the construction of laws similar to the law that exempts from its operation those who were engaged in the general practice of medicine for five years prior to January, 1875, there have been numerous decisions, all holding to their constitutionality. Medical Assn. vs. Schrader, 55 N. W. Rep., 24; Gee Wo vs. The State, 54 N. W. Rep., 513; The State vs. Carey, 30 Pac. Rep., 729; The State vs. Randolph, 31 Pac. Rep., 201; The State vs. Van Doran, 14 S. E. Rep., 32; Logan vs. The State, 5 Texas Cr. App., 306.

We can see no conflict between the statute and the constitution. The conflict set up is, that the statute provides that the board of examiners must be graduates of some medical college, recognized by the "American Medical Association," and that this Association is composed entirely of adherents to the allopathic school of medicine. This court can not possibly know that to be a fact, and of course can not go out into the field of speculation in connection with that subject.

Coming to the main question, we are of the opinion that the petition did not show appellant to be in a position to recover for his services as a physician. The laws of Texas make it a grave misdemeanor to practice medicine without obtaining a certificate of professional qualification from some authorized board of medical examiners, or without having a diploma from some accredited medical college, chartered by the Legislature of the State, or its authority, in which the same is situated; and even after the certificate or diploma is obtained, it is a penal offense not to have filed the certificate or diploma for record with the clerk of the District Court of the county in which the physician resides. The civil statute, article 3632, provides, that every applicant to practice medicine in Texas, whether he has a medical diploma or not, must be examined by the board of examiners, and in the exceptions none are mentioned but those qualified to practice medicine under the act of May 16, 1873, those who had been regularly engaged in the general practice of medicine in Texas for five years prior to January 1, 1875, and females who follow the practice of midwifery strictly as such. Rev. Stats., article 3637.

While a physician must violate the civil statute to practice medicine, even when he has a diploma, without first obtaining a certificate from a board of medical examiners, yet under the criminal statute it would seem that a diploma from a chartered accredited medical college will exempt from punishment under article 396, but under article 398 it is a violation of the law to fail to record either.

The petition alleges that appellant had a diploma from "a reputable American medical college," but there is no allegation that it had ever been recorded as required by law.

The laws, both civil and criminal, were passed for the protection of the public against charlatans and quacks who might engage in the practice of medicine. It is a responsible and honorable profession, whose members are brought into the closest contact with every family in the land, and to whom necessarily the most sacred matters of life and death must be confided. Society has a right to demand the closest scrutiny of the credentials of those in whose hands the health and lives of its members must sooner or later be placed. In answer to this demand the laws of this State were passed. It not only protects society, but the profession, from the odium that would attach by reason of unworthy and incompetent members. To practice medicine without having obtained the certificate and without having it duly recorded, is in violation of the civil statute, and this failure can not be excused on the ground that no board of medical examiners had been appointed. A violation of law can not be excused on the ground that some one else has violated it; and if there was no medical board in Bexar county, as required by law, appellant should have applied to some other board for examination, or at least have had his diploma recorded, in order that others might see it, and ascertain whether it was from some "reputable American college." The petition, in alleging the possession of a diploma, was defective under the criminal statute, in not stating that it was issued by a chartered accredited college.

The question before us has not, we believe, been ever directly decided in this State, but it has been before the Supreme Courts of a number of the States, and they uniformly hold that a physician practicing medicine in violation of law can not recover for fees for his services. In New York it is held that any person who practices as a physician without obtaining and filing the certificate mentioned in the statute, can not recover compensation for his services, although the statute does not expressly forbid the recovery of compensation. Fox vs. Dixon, 12 N. Y., 267. The ground of this decision was that it was a violation of a penal statute to practice without the certificate.

In Tennessee it is held, that a physician practicing without getting the certificate required by statute can not recover upon an account for medical services rendered, and the ground of this decision is thus stated: "When a statute has for its manifest purpose the promotion of some object of public policy, and prohibits the carrying out of a profession, occupation, trade or business except in compliance with the statute, a contract made in violation of such statute can not be enforced." Haworth vs. Montgomery, 18 S. W. Rep., 399.

In California it is said, that the statute making it a misdemeanor to practice medicine without a certificate from a medical board, remuneration for services rendered while the physician had no such certificate can not be recovered. Gardner v. Tatum, 22 Pac. Rep., 880; Roberts v. Levy, 31 Pac. Rep., 570.

In Kansas it is held, that a physician practicing medicine without complying with the law can not recover pay for services rendered, the decision being based on the ground that it would be contrary to public policy to permit the recovery. Underwood v. Scott, 23 Pac. Rep., 942. The Kansas statute, as does ours, provides that no person, save no exceptions, shall be permitted to practice medicine in any of its branches or departments without having first obtained and recorded a certificate of qualification from some authorized board of medical examiners.

In North Carolina it is held, that a contract under which a physician, not having a license as required by law, rendered services was void, and he could not recover pay for the same. This decision was under a law that provides that "No person shall practice medicine or surgery, nor any of the branches thereof, nor in any case prescribe for the cure of diseases, for fee or reward, unless he shall have been first licensed so to do in the manner hereinafter provided; provided, that no person who shall practice in violation of this chapter shall be guilty of a misdemeanor." In rendering the opinion the court said: "The performance of such service for fee or reward was absolutely prohibited by the statute, and the contract was therefore void in its inception. It is immaterial whether the act was malum in se or merely malum prohibitum. Ruffin, Chief Justice, in Sharp v. Farmer, 4 Devlin & Battle, 122, said, that the distinction between these 'was never sound, and is entirely disregarded; for the law would be false to itself if it allowed a party through its tribunals to derive advantage from a contract made against the intent and express provisions of the law.' " Puckett v. Alexander, 8 S. E. Rep., 767. This case emphasizes the principle that the mere fact that an act is not punishable under the penal code, but is in violation of a civil statute, would render the contract void.

We are of the opinion that the contract shows on its face that appellant was practicing medicine without having complied with the terms of the law as embodied in the title 73, articles 3625 to 3638 inclusive, and that it would be contrary to public policy to enforce his contracts in the courts of the country. The law was passed for the public good, both in protecting it from acts of empiricism, and at the same time shielding a noble profession from unworthy and unskillful members. Courts can not countenance violations of the law by enforcing contracts made in defiance of it.

We see no error in the judgment of the District Court, and it is affirmed.

Delivered February 28, 1894. *Affirmed.*

