# Erratum

**Published:** 1896-02

**Authors:** 


**Erratum.**—In our last issue it was stated that Dr. W. F. Starley, Jr., of Tyler, had been appointed resident student at the Sealy hospital. That was a mistake; Dr. Starley was elected by the Faculty of the Texas Medical College to the responsible position of Resident Physician and Superintendent,—quite a compliment and worthily bestowed.

